# Early Treatments of Fragile Children with COVID-19—Results of CLEVER (Children COVID Early Treatment), a Retrospective, Observational Study

**DOI:** 10.3390/v15010192

**Published:** 2023-01-10

**Authors:** Chiara Minotti, Daniele Mengato, Marica De Pieri, Sabrina Trivellato, Andrea Francavilla, Costanza Di Chiara, Cecilia Liberati, Raffaele Mattera, Alessandra Biffi, Carlo Giaquinto, Francesca Venturini, Daniele Donà

**Affiliations:** 1 Division of Pediatric Infectious Diseases, Department of Women’s and Children’s Health, University of Padua, Via Giustiniani, 3, 35128 Padua, Italy; 2 Hospital Pharmacy Department, University Hospital of Padua, Via Giustiniani, 2, 35128 Padua, Italy; 3 Unit of Biostatistics, Epidemiology and Public Health, Department of Cardiac, Thoracic, Vascular Sciences and Public Health, University of Padova, Via Loredan, 18, 35131 Padova, Italy; 4 Division of Pediatric Hematology, Oncology and Stem Cell Transplant, Department of Women’s and Children’s Health, University of Padua, Via Giustiniani, 3, 35128 Padua, Italy

**Keywords:** COVID-19, off-label, antivirals, monoclonal antibodies, children

## Abstract

(1) Background: SARS-CoV-2 infection is notably mild in children, though comorbidities may increase the risk of hospitalization and may represent a risk for increased disease severity. There is an urgent need for targeted therapies with an acceptable efficacy and safety profile. To date, most of the medicines for COVID-19-specific treatment are prescribed off-label for children due to a lack of clinical trials and consequent evidence in this population. (2) Methods: This was a retrospective, observational study investigating the safety of treatments for the prevention of severe COVID-19 in fragile pediatric patients who received monoclonal antibodies and antivirals for mild-to-moderate symptoms between December 2021 and July 2022. (3) Results: Thirty-two patients were included. Monoclonal antibodies were prescribed to 62%, intravenous antivirals to 22%, and oral antivirals to 16% of children. Sotrovimab was the most frequently prescribed drug among monoclonal antibodies and overall (59%). The second most prescribed drug was remdesivir (22%). No severe adverse drug reaction was reported. There was no progression to severe disease and no death cases due to COVID-19 or drug administration. At drug-type stratification, resolution of symptoms and swab positivity time showed no difference between the two groups at 7 and 28 days. Off-label prescriptions were 84% overall, and in similar proportions between the two groups. (4) Conclusions: in this small sample, antivirals seemed safe and showed no differences in efficacy as compared to MAbs for the early treatment of COVID-19 in fragile children, thus representing a valuable choice, even when administered off-label.

## 1. Introduction

Since the beginning of the COVID-19 pandemic in March 2020, subsequent waves of infection still challenge health resources and services globally. The pediatric population usually presents no or mild symptoms, with a relatively low risk of developing severe SARS-CoV-2 disease, including Delta and Omicron variants [1,2,3]. Nonetheless, many children require hospital admission, especially those with comorbidities, as exposure to SARS-CoV-2 is almost universal, thus contributing to the hospitalization burden globally [4,5,6]. In the early stage of the pandemic, children have not been included in the clinical trials for COVID-19 treatment development, with consequently limited evidence in this age group. For this reason, many of these drugs, such as antivirals and monoclonal antibodies (MAbs), are used off-label in children, especially those under 12 years of age and weighing less than 40 kg (https://www.who.int/publications/i/item/WHO-2019-nCoV-therapeutics-2022.4, accessed on 1 September 2022) [7,8]. Moreover, fragile children, such as immunosuppressed patients, transplant recipients, or with onco-hematological, pulmonary, or cardiac comorbidities, might benefit from early treatment as soon as SARS-CoV-2 positivity is discovered through nasopharyngeal swab, even in the absence of symptoms.

We aim to report our experience with the early treatment of COVID-19 with antivirals and MAbs in fragile pediatric patients from a single University Hospital in Northern Italy.

## 2. Materials and Methods

### 2.1. Study Design, Case Definition, Inclusion and Exclusion Criteria

We designed a retrospective observational study (CLEVER, Children COVID Early Treatment), to investigate the real-life safety of treatments to prevent severe COVID-19 in fragile pediatric patients. In our analysis, we included all patients aged 0–18 years, afferent to the Department of Women’s and Children’s Health of the University Hospital of Padova (Veneto Region, Italy), who received, between December 2021 and July 2022, at least one of the following medications for the early treatment of COVID-19:

- oral antiviral drugs (nirmatrelvir/ritonavir and molnupiravir);
- intravenous antiviral drugs (remdesivir);
- MAbs (sotrovimab and bamlanivimab–etesevimab as the only available options).

Each patient with a SARS-CoV-2 infection confirmed by polymerase chain reaction (PCR) had to be asymptomatic or present mild/moderate symptoms AND present baseline clinical conditions with a high risk of developing a severe disease to be eligible for treatment.

These risk factors include the following:

- Primary immunodeficiencies;
- Secondary immunodeficiencies, including transplant recipients, and with special regard to oncohematology patients being treated with myelosuppressive/immunosuppressive drugs, myelosuppressive drugs, or less than six months after discontinuation of treatment;
- autoimmune diseases;
- sickle cell anemia;
- congenital metabolic disorders;
- neurodevelopmental diseases;
- chronic respiratory diseases; with technological device dependence (e.g., individuals with tracheotomy, gastrostomy, etc.).

We included all eligible patients, regardless of their weight. For all patients with a body weight over 40 kg, the standard dosage expected for the adult patient was used as follows:

- sotrovimab: 500 mg/day;
- bamlanivimab + etesevimab: 700 mg + 1400 mg/day;
- remdesivir: 200 mg/on day 1, followed by 100 mg/day the next 2 days;
- nirmatrelvir/ritonavir: 300 mg + 100 mg twice daily for 5 days;
- molnupiravir: 800 mg twice daily for 5 days.

For patients weighing less than 40 kg, dose adjustment formulas were applied according to the literature and international guidelines [9,10,11]. No patients in this weight range were treated with oral antivirals, so no revision of the dosing schedule was necessary for these drugs.

The choice of a specific drug for treatment depended on the available molecule at our facility at the time, on the predominant variant(s) for MAbs, and on possible known interactions with the basal therapy. Patients whose parents did not consent to the study and those who had been experiencing symptoms for more than five days were excluded from the analysis.

### 2.2. Data Collection

For each patient, clinical, demographic, diagnostic, and prescription data were manually collected from electronic medical records and stored in a password-protected data collection sheet stored on a secured server at the University of Padova. Privacy was guaranteed by assigning each patient a unique, anonymous study code and not collecting personally identifying data.

Patients were divided into two groups, according to the administered drug: 1. Antivirals and 2. MAbs. The comparability of groups was checked in terms of gender, age, and weight distribution. Endpoints for comparison among groups included symptoms resolution at 7 and 28 days, time to PCR swab negativization and evaluation of adverse events at 7 and 28 days after drug administration.

The following variables, selected a priori, were tested as determinants of outcome: patient sex, age, and ongoing immunosuppressive regimen; COVID-19 vaccination status; steroid therapy; comorbidities; first positive swab date; COVID-19 symptoms and targeted therapy; off-label drug prescription status; basal serum creatinine and transaminase; treatment start and discontinuation date; complete targeted therapy status; outcome at 7 and 28 days; serious adverse events; mortality; mechanical ventilation; non-invasive respiratory support; oxygen need; and date of the first negative swab.

### 2.3. Outcomes

The outcomes of the study are (I) the evaluation of serious adverse events at 7 and 28 days after drug administration, (II) the resolution of clinical symptoms at day 7 and 28 after therapy administration, defined as resolution of febrile episodes and/or respiratory symptoms and/or other COVID-related symptoms, and (III) time to PCR swab negativization.

### 2.4. Ethical Considerations

Each treatment, before being administered, required informed consent from the patient’s caregivers.

The investigations were carried out following the rules of the Declaration of Helsinki of 1975 (https://www.wma.net/what-we-do/medical-ethics/declaration-of-helsinki/, accessed on 1 September 2022), revised in 2013. This study was approved by the Ethical Committees of Padova University Hospital (n. 0065700).

### 2.5. Data Analysis

Continuous data were reported as the median and interquartile range (IQR). Categorical variables were reported as frequencies and percentages.

## 3. Results

A total of 32 consecutive patients (18, 56% males) were eligible and included in the study. The median (IQR) age was 8.5 (5.4, 10.7) years, and the median weight was 24 (17, 42) kg. A total of 22 patients had an underlying oncohematological disease (69%); 4 underwent transplantation (12%); and 6 had other chronic conditions (congenital metabolic disorders, autoimmune diseases, primary immunodeficiencies, technological device dependence; 19%).

None of the patients had received the COVID-19 vaccine. A total of 19 children (59.3%) were on polytherapy, and 13 (41%) were receiving steroids. A total of 11 children (34%) were asymptomatic, while the remaining 21 (66%) had mild symptoms. MAbs were prescribed to 20 (62%) children, intravenous antivirals to 7 (22%), and oral antivirals to 5 (16%) children. Sotrovimab was the most frequently prescribed drug among MAbs and overall (19 patients, 59%). The second most prescribed drug was remdesivir (7 patients, 22%), followed by nirmatrelvir–ritonavir (3 children, 9.4%), molnupinavir (2 children, 6.2%), and bamlanivimab–etesevimab (one patient, 3.1%). Off-label prescriptions were 84% (27/32) overall.

Baseline characteristics are fully described in Table 1. A total of 12 patients received an antiviral drug, while 20 patients received an MAb. In the antiviral group, remdesivir was chosen in more than half of the cases (7/12 patients, 58%). In the MAbs group, almost all the patients received sotrovimab (19/20, 95%).

The two groups showed similar characteristics, as displayed in Table 2. Notably, all SOT recipients received an MAb, while oncohematological patients received, in similar proportions, antivirals and MAbs (10 vs. 12, 60% vs. 83%, respectively). Baseline features for specific drugs are described in Table S1 Supplementary.

No severe adverse drug reaction was reported in either group, and there were no death cases due to COVID-19 progression or due to drug administration. Three deaths were registered in total, all related to the patients’ underlying disease. Three patients (9%) required oxygen supplementation, and one (3%) required non-invasive ventilation during SARS-CoV-2 infection. None required intubation and mechanical ventilation. A mild and transient transaminase elevation was described as the only encountered adverse reaction.

**Figure 1:**
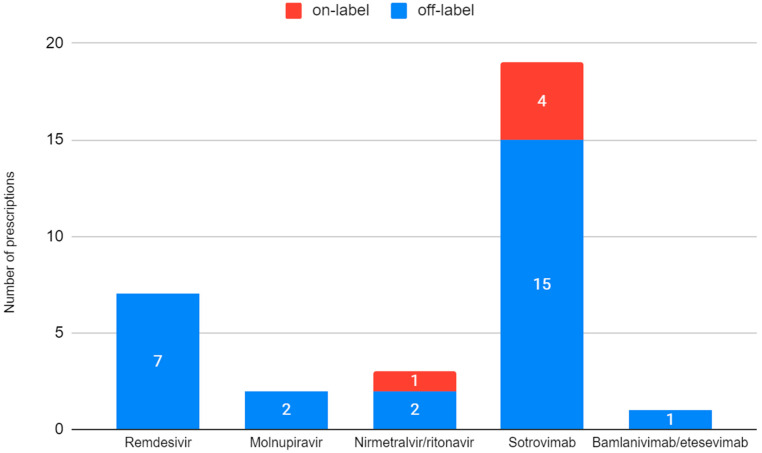
Off-label prescriptions according to the administered drugs.

The proportion of off-label prescriptions was elevated in both groups, with 4/20 patients with an on-label prescription (20%) in the MAbs group, who received sotrovimab, and 1 patient out of 12 (8%) in the antiviral group, who received nirmatrelvir–ritonavir (Table S1 Supplementary, Figure 1).

One patient in the antivirals group was lost to follow-up at seven days, and one was lost at 28 days in the MAbs group. Resolution of symptoms/asymptomatic status and swab positivity duration was similar between the two groups at 7 and 28 days, with a slightly inferior duration of positivity in the MAbs group.

**Figure 2:**
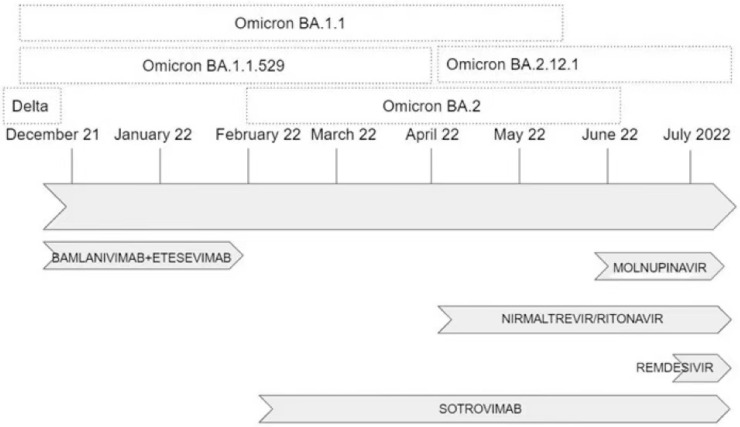
Timeline of variants/subvariants and administration of specific drugs over the study period.

Figure 2 shows the timeline of variants/subvariants and the administration of specific drugs over the study period.

## 4. Discussion

To the best of our knowledge, this study is the first to present evidence on the safety of both antivirals and MAbs, mainly prescribed off-label, for the precocious treatment of fragile children. The administration of antivirals and MAbs led to no severe adverse events in our population. Only a mild and transient elevation in transaminase level was reported in some patients. Despite the limitations due to the small sample size, these are relevant findings in an international landscape where optimal dosing regimens and safety data are still unknown for children. Most importantly, there were no cases of progression to severe disease, requiring mechanical ventilation, and death due to COVID-19 or as a result of the administration of the drugs. Before treatment administration, more than half of the patients presented mild symptoms, while the rest were asymptomatic. Considering the risk–benefit ratio of treating a selected population of fragile pediatric patients, completely asymptomatic or with mild symptoms, we decided in favor of treatment in light of the importance of basal comorbidities and the potential risk of progression to severe COVID-19. A total of 38% percent of patients were treated with either oral or intravenous antivirals, while 62% received MAbs, according to the available molecules at the time. Remdesivir and sotrovimab were the most widely used medicines among antivirals and MAbs, respectively, being largely available at our facility. Considering the other outcomes, the duration of SARS-CoV-2 swab positivity and the proportion of symptomatic patients were similar between the 2 groups at 7 and 28 days.

Since the beginning of the pandemic, it was clear that cases of COVID-19 in the pediatric population were mostly paucisymptomatic or asymptomatic, generally with a lower risk of hospitalization [2,12]. Concerns have been raised about the subpopulation of fragile children, often already-admitted, contracting SARS-CoV-2, or being hospitalized for COVID-19. If immunosuppressed children have similar clinical involvement and outcomes as the pediatric population [3,13], the presence of other comorbidities, in general, represents a higher risk for hospitalization. The use of COVID-19 treatment is therefore especially addressed to this frail group, and almost universally off-label, with the risk of safety issues.

Doses for COVID-19-specific treatment are derived from studies in the adult population, sometimes also including adolescents, but seldom in individuals younger than 12 years and weighing less than 40 kg. Indeed, randomized controlled trials (RCTs) have not systematically included the pediatric population so far, with scarce evidence on efficacy, safety, and optimal doses [4,7,14]. Moreover, ethical concerns have risen around the construction of RCTs for COVID-19 antivirals and MAbs in children, arguing about the possibly unethical nature of trials, as severe presentations are rare [15]. Therefore, the risks of clinical trials in this population must be weighed against the benefits to the participants and the general pediatric population [16]. Nonetheless, with the evolving spectrum of COVID-19, the need for evidence on the efficacy and safety of COVID-19 drugs for children is imperative, especially to minimize morbidity and mortality related to severe disease, protecting the most vulnerable patients and limiting the harm related to the use of potentially ineffective molecules [16]. Notably, there is an ongoing trial on tocilizumab (RECOVERY NCT04381936) also including children.

*Evidence on antivirals*

Remdesivir was the first antiviral to be authorized for COVID-19 treatment in October 2020. The Food and Drug Administration (FDA) approved remdesivir for hospitalized children ≥12 years and ≥40 kg, regardless of disease severity, then provided emergency use authorization (EUA) for patients weighting between 3.5 and 40 kg who are admitted and at higher risk for evolution to severe disease. It has not been approved in patients younger than 12 years. There is evidence of compassionate use of remdesivir in children with severe and non-severe COVID-19, especially with underlying complex medical conditions and/or infants, with clinical benefit and a good safety profile, similarly to what emerged from our results [8,17]. Remdesivir is recommended intravenously for 3–5 days for children with a positive SARS-CoV-2 PCR test dating back to the previous 3–4 days [7]. In our study, the currently available indications were followed for its use on our population.

Nirmatrelvir–ritonavir was given EUA by the FDA over 12 years and 40 kg, in case of mild-to-moderate COVID-19 and high risk for severe disease. Evidence is available following the results of EPIC-HR RCT on unvaccinated adults, showing a lower risk of hospitalization or death after administration within five days [18]. It is an inhibitor of cytochrome (CYP) P450 3A, to be considered in the case of polytherapy regimens, with other drugs that may induce or be metabolized by the same pathway, for risk of lower drug concentrations and a lesser response. EPIC-HR RCT in its phases 2 and 3 is underway on a population of patients of ages between 6 and 17 years [7].

Molnupinavir has a conditional recommendation in patients with non-severe COVID-19, at the highest risk of hospitalization, and is off-label in children. RCTs are ongoing, evaluating efficacy and safety in adults [19].

Nirmatrelvir–ritonavir and molnupiravir were less used in our population. This may be due to drug formulation issues, as these drugs are available as tablets, which may not be easily administered to younger children. Moreover, ritonavir is no longer effective if the tablet is split or crushed. Currently, there are no published studies in the literature providing evidence on their use in fragile children with COVID-19.

*Evidence on MAbs*

As regards to MAbs, notably, the experience with other molecules, such as palivizumab for respiratory syncytial virus prophylaxis, and tocilizumab, already licensed for juvenile idiopathic arthritis in patients over two years of age, is already well-established. This background may have supported the use of other MAbs in children, even off-label and without well-established safety data [20]. Among available evidence, data from a recent study by Romani et al. already reported the safety of MAbs in children at high risk of developing severe COVID-19, along with a case report on the use of sotrovimab in patients under 12 years [21,22].

Almost all children in our population received sotrovimab, which binds to the preserved epitope on the virus’s spike (S) protein receptor-binding domain [23]. It obtained EUA by the FDA, and its use was approved by the EMA in patients ≥12 years and ≥40 kg at increased risk of hospitalization and/or death from COVID-19, not requiring oxygen supplementation. Its neutralizing activity against the widespread Omicron BA.2 subvariant was then proved to be reduced, so it is no longer recommended where this variant is dominant. Among recent evidence, there is a case series of 5 children younger than 12 years old safely receiving sotrovimab for a high risk of progression to severe COVID-19 [24]. Despite the fact the sample was smaller than the one in this study, sotrovimab administration was similarly safe.

Bamlanivimab–etesevimab had received EUA by the FDA for treatment of mild-to-moderate COVID-19 in pediatric patients as well, including neonates, with positive results of SARS-CoV-2 viral testing, and at high risk for progression to severe disease. Former evidence from 2021 included a phase 3 trial with 1:1 randomization of a cohort of ambulatory patients (including adolescents > 12 years) with mild or moderate COVID-19, who were at high risk for progression to severe disease, to receive bamlanivimab–etesevimab vs. a placebo. This led to a lower incidence of COVID-19-related hospitalization and death than the placebo and accelerated the reduction of the SARS-CoV-2 viral load [25]. Since January 2022, however, its use is no longer recommended due to its inactivity against the Omicron variant [9].

### 4.1. Limitations of the Study

A limitation of our study is represented by its retrospective nature and the small numerosity of our sample, which makes it difficult to apply our findings to the general population of fragile children. Moreover, due to the study design, the results were not evaluated in comparison to a population of healthy children with no risk factors and COVID-19 infection, nor to a similar population receiving no treatment. Lastly, the choice of the molecule was limited and determined by the available medicines at the time, or, as regards to MAbs, by the currently circulating SARS-CoV-2 variant.

### 4.2. Conclusions

CLEVER is one of the first studies providing evidence on the off-label use of both antivirals and MAbs in children. In this small sample, antivirals and MAbs showed a good safety profile, thus representing a valuable choice, even when administered off-label. Rigorous and well-designed RCTs of such drugs in children are needed, to determine the efficacy and safety of COVID-19 treatments, towards on-label use.

## Figures and Tables

**Table 1:** Baseline characteristics.

	*N* = 32 ^1^	
**Gender**		
*F*	14 (44%)	
*M*	18 (56%)	
**Age (years)**	8.5 (5.4, 10.7)	
**Age, categorical (years)**		
*≤2*	4 (12%)	
*>2*	28 (88%)	
**Weight (kg)**	24 (17, 42)	
**Baseline disease**		
*Oncohematological*	22 (69%)	
*Solid Organ Transplantation*	4 (12%)	
*Other*	6 (19%)	
**COVID-19 vaccination**		
*No*	32 (100%)	
**Steroid therapy**	13 (41%)	
**Polytherapy**	19 (59.3%)	
**COVID-19 symptoms**		
*Asymptomatic*	11 (34%)	
*Mild symptoms*	21 (66%)	
**Mechanical ventilation**	0 (0%)	
**Non-invasive ventilation**	1 (3%)	
**Oxygen requirement**	3 (9%)	
**Type of drug**		
*Antiviral*	12 (38%)	
*MAbs*	20 (62%)	
**COVID-19 drug**		
*Bamlanivimab*–*Etesevimab*	1 (3.1%)	
*Molnupiravir*	2 (6.2%)	
*Nirmatrelvir* *–* *Ritonavir*	3 (9.4%)	
*Remdesivir*	7 (22%)	
*Sotrovimab*	19 (59%)	
**Off-label prescription**	27 (84%)	
**Serum creatinine (μmol/L)**	36 (26, 56)	
**AST (U/L)**	48 (30, 69)	
**ALT (U/L)**	42 (26, 78)	
**Symptoms after 7 days**	**Antiviral, *N* = 12 ^1^**	**MAbs, *N* = 20 ^1^**
*Asymptomatic*	9 (82%)	18 (94.8%)
*Still Symptoms*	2 (18%)	1 (5.2%)
**Positivity after 7 days**		
*Negative*	0 (0%)	1 (5.2%)
*Positive*	10 (100%)	16 (94.8%)
**Symptoms after 28 days**		
*Asymptomatic*	10 (100%)	16 (94%)
*Still symptoms*	0 (0%)	1 (5.9%)
**Positivity after 28 days**		
*Negative*	6 (60%)	11 (55%)
*Positive*	4 (40%)	5 (33%)
**Severe ADR**		
*No*	12 (100%)	20 (100%)
**Death for other causes**	1 (8.3%)	2 (10.5%)
**Lost to follow-up at 7 days**	1 (8.3%)	0 (0%)
**Lost to follow-up at 28 days**	0 (0%)	1 (5%)

Legend: ^1^ n (%); Median (IQR); F: female; M: male; MAbs: monoclonal antibodies; AST: aspartate aminotransferase; ALT: alanine aminotransferase; ADR: adverse drug reaction.

**Table 2:** Baseline characteristics according to the administered drug.

		Type of Drug	
	Overall, *N* = 32 ^1^	Antiviral, *N* = 12 ^1^	MAbs, *N* = 20 ^1^
**Gender**			
*F*	14 (44%)	4 (33%)	10 (50%)
*M*	18 (56%)	8 (67%)	10 (50%)
**Age (years)**	8.5 (5.4, 10.7)	8.5 (5.6, 11.7)	8.5 (5.2, 10.3)
**Age, categorical (years)**			
*≤2*	4 (12%)	1 (8.3%)	3 (15%)
*>2*	28 (88%)	11 (92%)	17 (85%)
**Weight (kg)**	24 (17, 42)	23 (21, 45)	24 (16, 31)
**Baseline disease**			
*Oncohematological*	22 (69%)	10 (83%)	12 (60%)
*Solid Organ Transplantation*	4 (12%)	0 (0%)	4 (20%)
*Other*	6 (19%)	2 (17%)	4 (20%)
**COVID-19 vaccination**			
*No*	30 (100%)	11 (100%)	19 (100%)
**Steroid therapy**	13 (41%)	4 (33%)	9 (45%)
**Polytherapy**	15 (47%)	6 (50%)	9 (45%)
**COVID-19 symptoms**			
*Asymptomatic*	11 (34%)	6 (50%)	5 (25%)
*Mild symptoms*	21 (66%)	6 (50%)	15 (75%)
**Off-label prescription**	27 (84%)	11 (92%)	16 (80%)
**Creatinine (μmol/L)**	36 (26, 56)	31 (22, 40)	39 (28, 56)
**AST (U/L)**	48 (30, 69)	52 (34, 74)	45 (31, 68)
**ALT (U/L)**	42 (26, 78)	45 (28, 87)	41 (26, 71)

Legend: ^1^ n (%); Median (IQR); MAbs: monoclonal antibodies; F: female; M: male; AST: aspartate aminotransferase; ALT: alanine aminotransferase.

## Data Availability

The datasets supporting the conclusions of this article are available upon a reasonable request to the corresponding author.

## Supplementary Materials

The following supporting information can be downloaded at: https://www.mdpi.com/article/10.3390/v15010192/s1, Table S1: Patients’ basal features according to specific drugs.
