# Supplementary material for: Early Treatments of Fragile Children with COVID-19—Results of CLEVER (Children COVID Early Treatment), a Retrospective, Observational Study
Source: Viruses. 2023 Jan 10;15(1):192. doi: 10.3390/v15010192 (PMC9867507; doi:10.3390/v15010192)
Supplement: Supplementary file 1 [file viruses-15-00192-s001.zip › viruses-2132305-supplementary.pdf]

Supplementary Material

Table 1 Supplementary. Patients' basal features according to specific drugs.

	Bamlanivimab-Etesevimab	Molnupinavir	Nimatrelvir - Ritonavir	Remdesivir	Sotrovimab
**Patient n. (%)**	1/32 (3)	2/32 (6.2)	3/32 (9.3)	7/32 (21.8)	19/32 (59.7)
**Gender**					
F **n. (%)**	1 (100)	1 (50)	1 (33)	2 (28.5)	10 (52.6)
M **n. (%)**	0	1 (50)	2 (67)	5 (71.5)	9 (47.4)
**Age (years)[1,2]**	6	9.7-10.3[1]	15-18[1]	2.5 (4.25,6.75)[2]	8.44 (4.88,10.83)[2]
**Age, categorical (years)[1,2]**					
<=2	0	0	0	1	3
>2	1	2	3	6	16
**Weight (kg)**	24.2	42.5-45[1]	59-65[1]	15.5 (6.5,22)[2]	23.5 (6.5,30)[2]
**Baseline disease n. (%)**					
Oncohematological	1 (100)	2 (100)	2 (66.6)	6 (85.7)	11 (57.8)
Solid Organ Transplantation	0	0	0	0	4 (21.1)
Other	0	0	1 (33.4)	1 (14.3)	4 (21.1)
**COVID-19 vaccination n. (%)**					
No	1 (100)	2 (100)	3 (100)	7 (100)	19 (100)
**Steroid therapy**	1(100)	2 (100)	0	2 (25.8)	8 (42)
**Polytherapy**	1(100)	2 (100)	3 (100)	2 (25.8)	11 (57.8)
**COVID-19 symptoms n. (%)**					
Asymptomatic	1 (100)	1 (50)	2 (66.6)	3 (42.8)	4 (21)
Mild symptoms	0	1 (50)	1 (33.4)	4 (57.2)	15 (79)
**Mechanical ventilation n. (%)**	0	0	0	0	0
**Non-invasive ventilation n. (%)**	1 (100)	0	0	0	0
**Oxygen requirement n. (%)**	1 (100)	0	0	1 (14.2)	1 (5.2)
**Off-label prescription n. (%)**	1 (100)	2 (100)	2 (66.6)	7 (100)	15 (78.9)
**Serum creatinine (μmol/L)**	15	23-118[1]	35-60[1]	18 (30,31)[2]	40.5 (29,56.7)[2]
**AST (U/L)**	69	13-24[1]	28-71[1]	67 (47,89)[2]	42 (27.5,66)[2]
**ALT (U/L)**	39	6-35[1]	39-125[1]	70 (33.5,87)[2]	44 (24,75.7)[2]
**Symptoms after 7 days n. (%)**					
Asymptomatic	1 (100)	2 (100)	1 (33.4)	5 (71.4)	17 (89.4)
Still Symptoms	0	0	1 (33.4)	1 (14.2)	1 (5.2)
**Positivity after 7 days n. (%)**					

Negative	0	0	/	/	1 (5.2)
Positive	1 (100)	2 (100)	2 (66.6)	6 (85.7)	15 (78.9)
**Symptoms after 28 days** n. (%)					
Asymptomatic	0	2 (100)	2 (66.6)	6 (85.7)	16 (84.2)
Still symptoms	1 (100)	0	/	/	/
**Positivity after 28 days** n. (%)					
Negative	0	1 (50)	1 (33.4)	4 (57.1)	11 (57.8)
Positive	1 (100)	1 (50)	1 (33.4)	2 (28.5)	4 (21)
**Severe ADR** n. (%)					
No	1 (100)	2 (100)	3 (100)	7 (100)	19 (100)
**Death for other causes** n. (%)	0	0	0	1 (14.2)	2 (10.5)
**Lost at follow-up at 7 days**	0	0	1 (33.)	0	0
**Lost at follow-up at 28 days**	0	0	0	0	1 (5.2)

1 Range, 2 Median (Interquartile range)
